# Iron Oxide Nanoparticles for Magnetically-Guided and Magnetically-Responsive Drug Delivery

**DOI:** 10.3390/ijms16048070

**Published:** 2015-04-10

**Authors:** Joan Estelrich, Elvira Escribano, Josep Queralt, Maria Antònia Busquets

**Affiliations:** 1 Departament de Fisicoquímica; Facultat de Farmàcia; Institut de Nanociència i Nanotecnologia (IN2UB), Universitat de Barcelona, 08028 Barcelona, Catalonia, Spain; E-Mail: mabusquetsvinas@ub.edu; 2 Departament de Farmàcia i Tecnologia Farmacèutica, Facultat de Farmàcia, Institut de Nanociència i Nanotecnologia (IN2UB), Universitat de Barcelona, 08028 Barcelona, Catalonia, Spain; E-Mail: eescribano@ub.edu; 3 Departament de Fisiologia, Facultat de Farmàcia; Institut de Nanociència i Nanotecnologia (IN2UB), Universitat de Barcelona, 08028 Barcelona, Catalonia, Spain; E-Mail: josepqueralt@ub.edu

**Keywords:** magnetic nanoparticles, magnetoliposomes, liposomes, gels, nanotechnology, controlled release, chemotherapy

## Abstract

In this review, we discuss the recent advances in and problems with the use of magnetically-guided and magnetically-responsive nanoparticles in drug delivery and magnetofection. In magnetically-guided nanoparticles, a constant external magnetic field is used to transport magnetic nanoparticles loaded with drugs to a specific site within the body or to increase the transfection capacity. Magnetofection is the delivery of nucleic acids under the influence of a magnetic field acting on nucleic acid vectors that are associated with magnetic nanoparticles. In magnetically-responsive nanoparticles, magnetic nanoparticles are encapsulated or embedded in a larger colloidal structure that carries a drug. In this last case, an alternating magnetic field can modify the structure of the colloid, thereby providing spatial and temporal control over drug release.

## 1. Introduction

Nanotechnology is a multidisciplinary branch of science that encompasses numerous fields of science and technology, including biomedicine, pharmaceutics, agricultural sciences, environmental sciences, advanced materials science, chemistry, physics, electronics, information technology, and others [1]. In biomedicine, nanoscale materials are of special importance due to their size being compatible with cells (10–100 μm), viruses (20–450 nm), proteins (5–50 nm) and genes (2 nm wide by 10–100 nm long). Nanomaterials are small enough to move inside the body without disrupting normal functions and can access places that are inaccessible to other materials [2]. Cells react in the presence of nanomaterials, and these reactions can induce changes in cells, including cell growth or death [3].

One of the most commonly-used nanoscale materials are magnetic nanoparticles (MNPs): a type of core/shell nanoparticle structure that consists of a magnetic core encapsulated in an organic or a polymeric coating. Without a coating, MNPs have hydrophobic surfaces with large surface-to-volume ratios and a propensity to agglomerate [4]. MNPs, which exhibit a variety of unique magnetic phenomena that are drastically different from those of their bulk counterparts, are generating significant interest, since their properties can be utilized in a variety of applications, ranging from storage media for magnetic memory devices to probes and vectors in the biomedical sciences [5].

The large surface-to-volume ratio of MNPs provides abundant chemically-active sites for biomolecule conjugation, thus allowing for precise design and engineering in order for them to meet their intended functions, such as long-lasting circulation in the blood stream, target specificity to lesion tissue, optical detectability and therapeutic delivery [6,7]. Their magnetic properties enable MNPs to be used in numerous applications related to drug and gene delivery, diagnostics and therapeutics. These applications can be classed as belonging to one or more of the following groups:

(1) Magnetic contrast agents for magnetic resonance imaging (MRI).
(2) Magnetic separation. For this, magnetic beads are functionalized with a biological or chemical agent known to bind to a specific target and mixed in a beaker with a solution believed to contain the target. After a specific time of contact between the beads and the solution, a permanent magnet placed alongside the solution beaker induces a magnetic moment and sets up a field gradient across the solution. The targets that have become bound to the magnetized beads are thus separated from the bulk solution.
(3) Immunoassays, where MNPs bound to primary or secondary antibodies are used to separate and quantify antigens.
(4) Hyperthermia agents, where the MNPs are selectively heated by application of a high-frequency alternating magnetic field.
(5) Magnetic vectors that can be directed by means of magnetic field gradients towards a certain location, as in the case of the targeted drug delivery.

**Figure 1:**
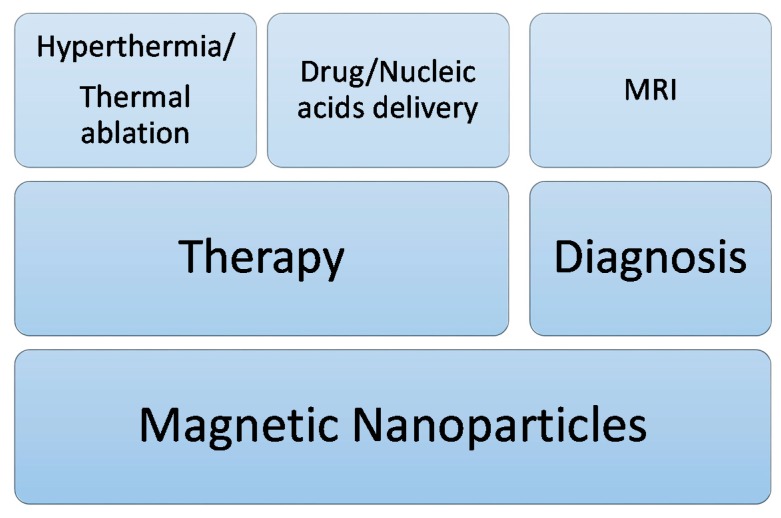
Biomedical applications of magnetic nanoparticles that rely on their magnetic properties.

Due to the fact that MNPs can be used in both diagnostic and therapeutic applications (Figure 1), they are excellent nanosystems with theranostic applications.

Here, we review recent advances in and problems with the development of targeted MNPs (*i.e*., magnetically-guided MNPs, MNPs for magnetofection and magnetically-responsive MNPs). Drug transport to specific targets and the ability to control the release of the cargo are two of the most avidly pursued goals in drug delivery research. The introduction of nucleic acids into cells is also a field of research with numerous therapeutic applications. This review focuses on the use of iron oxide nanoparticles (IONs) as particles that, after having been loaded with a drug or nucleic acids, can be directed to a chosen site via blood circulation by applying a localized external magnetic field. Furthermore, when IONs are encapsulated in some colloidal systems, such as liposomes or gels, the colloidal structure may become sensitive to an external magnetic field, and partial modification of the structure can result in the controlled release of the encapsulated drug.

## 2. Physical Principles of Magnetism

All materials are magnetic to some extent, with the degree depending on their atomic structure and the temperature. Electrons circulating around atomic nuclei, electrons spinning on their axes and rotating positively-charged atomic nuclei are all magnetic dipoles, also called magnetons. Altogether, these effects may cancel out, so that a given type of atom may not be a magnetic dipole. If they do not fully cancel out, however, the atom is a permanent magnetic dipole, as in the case of iron atoms. The strength of a magnetic dipole is called the magnetic dipole moment and may be thought of as a measure of the capacity of the dipole to align itself with a given external magnetic field.

When an external magnetic field (*H*) is applied to a material, the atomic dipoles tend to align themselves with the field, thereby causing a magnetic moment within the material. The quantity of magnetic moment per unit volume is defined as magnetization (*M*).

The relation between magnetization and the magnetic field is given by:

(1) M=χH

where *χ* is the volumetric magnetic susceptibility, which in SI units is dimensionless, and both *M* and *H* are expressed in A·m^−1^.

Magnetic materials may be conveniently classified in terms of their *χ* [8]. When the materials exhibit weak repulsion (negative susceptibility, with *χ* in the range −10^−6^ to −10^−3^), they are termed diamagnets. If the materials show small positive susceptibility (*χ* in the range 10^−1^ to −10^−6^), they are paramagnets; whereas a ferromagnet is a material that exhibits a large positive susceptibility. The magnetic properties of the first two categories do not persist if the external magnetic field is removed; while ferromagnetic materials have stable magnetic properties even after removal of the external field. A ferromagnet becomes a paramagnet above a temperature called the Curie temperature (*T*_C_): a temperature at which there is a change of the direction of the intrinsic magnetic moments. Below *T*_C_, the atoms lose their ordered magnetic moments and the material is paramagnetic.

**Figure 2:**
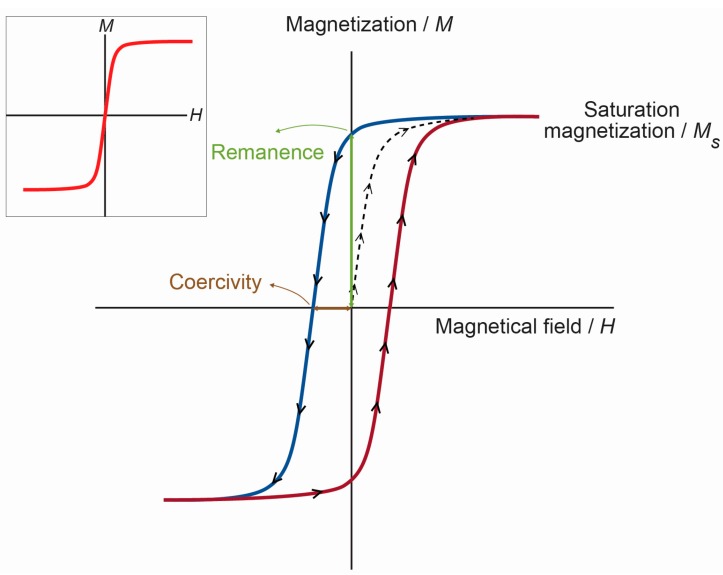
*M*-*H* curves for a ferromagnetic material. *M* is the magnetization of the material, and *H* is the external magnetic field. The arrows on the cycle indicate the direction when increasing or decreasing the field amplitude. Inset: *M*-*H* curve for a superparamagnetic material.

For diamagnets and paramagnets, the relationship *M* = *χH* is usually linear. In contrast, for ferromagnets, there is no one-to-one correspondence between *H* and *M*, and this relationship is not linear. If a paramagnet is demagnetized (*H* = *M* = 0) and the relationship between *M* and *H* is plotted for increasing levels of *H*, then *M* follows the initial magnetization curve (dashed line in Figure 2). This curve increases rapidly at first and then becomes asymptotic as it approaches magnetic saturation (*M*_s_). If *H* values are reduced monotonically, *M* follows a different curve (blue line in Figure 2). At *H* = 0, *M* is offset from the origin by an amount called the remanent magnetization, *M*_r_, which indicates the level of residual magnetism in the material. Therefore, the curve, of a sigmoidal shape, tends to a point where *M* = 0. This is called the point of coercivity on the curve. Therefore, the coercivity is the magnitude of the field that must be applied in the negative direction to bring the magnetization of the sample back to zero. As *H* increases in the negative direction, the material will again become magnetically saturated, but in the opposite direction. Increasing *H* in the positive direction again will return *H* to zero, and the curve returns to the saturation point (red line in Figure 2), where it completes the hysteresis loop. The width of the middle section is twice the coercivity of the material. The area of the hysteresis loop is related to the amount of energy dissipated upon reversal of the field.

**Figure 3:**
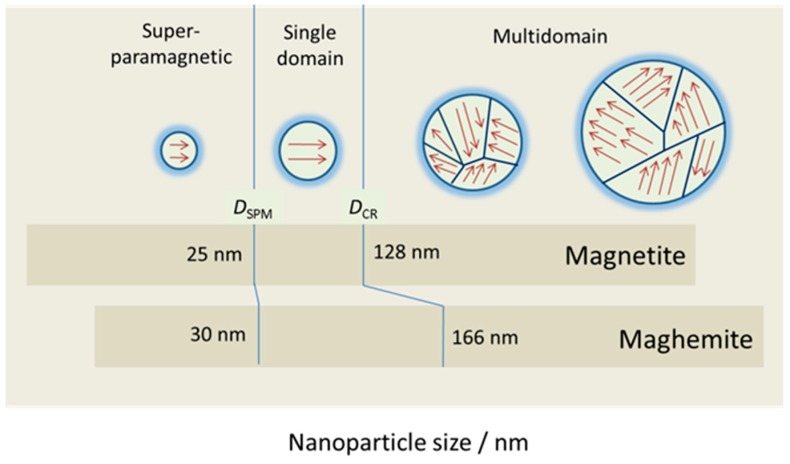
Magnetic regimes of magnetite and maghemite as a function of their size (superparamagnetic, single domain, multidomain).

Fundamental changes occur in the magnetic structure of macroscopic magnetically-ordered materials when their physical size is reduced [4]. In ferromagnetic materials, magnetons are associated in groups called domains. A magnetic domain refers to a volume of ferromagnetic material in which all magnetons are aligned in the same direction by exchanging forces (Figure 3). A bulk ferromagnet spontaneously subdivides into a multidomain structure to reduce the magnetostatic energy associated with a large stray field [9]. Within each domain, the magnetization does not vary; but between domains, there are relatively thin domain walls in which the direction of magnetization rotates from the direction of one domain to that of the other. The formation of the domain walls is a process driven by the balance between the magnetostatic energy, which increases proportionally to the volume of material, and the domain wall energy, which increases proportionally to the interfacial area between domains. When the size of a ferromagnetic material is reduced below a critical value, the so-called critical diameter *D*_CR_, more energy is required to create a domain wall than to support the external magnetostatic energy of the single domain state; the material becomes a single domain. The value of *D*_CR_ is typically a few tens of nanometers and depends on the material. The *D*_CR_ of a spherical particle is reached when the magnetostatic energy equals the interfacial energy.

A single domain particle is uniformly magnetized with all of the spins aligned in the same direction. The magnetization will be reversed by spin rotation, since there are no domain walls to move. Further reduction in size, below the superparamagnetic diameter *D*_SPM_, causes the thermal energy to exceed the energy barrier, which separates the two energetically-equivalent easy directions of magnetization (the easy axis is the preferred direction of the total magnetization of the dipoles of a given material), and the direction of the magnetization fluctuates randomly. Such a system is named a superparamagnet. The magnetic moments of individual crystallites compensate for each other, and the overall magnetic moment becomes null. When an external magnetic field is applied, the behavior is similar to paramagnetism, except that, instead of each individual atom being independently influenced by an external magnetic field, the magnetic moment of the crystallite aligns itself with the field. Consequently, superparamagnetic nanoparticles become magnetic in the presence of an external magnet, but revert to a non-magnetic state when the external magnet is removed. This is of paramount importance when these particles are introduced into living systems (e.g., in drug delivery), because, once the external magnetic field is removed, the magnetization disappears (they have negligible remanent magnetization and coercivity; see the inset in Figure 2), and thus, agglomeration (and the possible embolization of capillary vessels) is avoided [10]. The coercivity is zero for superparamagnets, but it increases in the single domain regimen and shows a peak with the development of multiple magnetic domains around *D*_CR_; then, as the particles reach the micrometer scale, the coercivity essentially becomes the same as that of bulk iron.

The shape of the loops in Figure 2 is determined in part by particle size: in larger particles, with a multidomain ground state, the hysteresis loop is narrow, since it takes relatively little field energy to make the domain walls move; while in smaller particles, there is a single domain ground state, which leads to a broad hysteresis loop.

Another important property of ferromagnets is magnetic anisotropy, which is the directional dependence of their magnetic moments. Whereas for a magnetically isotropic material (*i.e*., a superparamagnetic material), there is no preferential direction of the magnetic moment, a magnetically anisotropic material will align its magnetic moment in one direction, the so-called easy axis. Magnetocrystalline anisotropy is derived from spin-orbit coupling. The magnetocrystalline anisotropy constant (*K*) is a physical constant, which reflects the energy required to change the direction of magnetization from an easy to a hard axis (energetically unfavorable). The diameters mentioned above, *D*_SD_ and *D*_SPM_, can be worked out from *K*, the dimensionless hardness parameter (κ) and the exchange stiffness constant of the material.

Besides the size, the surface properties of MNPs are essential for their applications. Coating MNPs with a layer of a different material is an interesting method for modifying their surface properties. Several groups of coating materials are used to modify the surface chemistry of MNPs [11]:

(1) Organic polymers, such as dextran, chitosan, polyethylene glycol (PEG), polysorbate and polyaniline.
(2) Organic surfactants, such as sodium oleate and dodecylamine.
(3) Inorganic metals, such as gold.
(4) Inorganic, such as silica and carbon.
(5) Bioactive molecules and structures, such as liposomes, peptides and ligands/receptors.

Coated nanoparticles have major advantages over simple nanoparticles thanks to their enhanced properties, such as: (i) less cytotoxicity; (ii) increased dispersibility and biocompatibility; (iii) better conjugation with other bioactive molecules; and (iv) increased thermal and chemical stability [1].

For *in vivo* applications, MNPs need to exhibit high magnetic saturation. Furthermore, there may be other requirements depending on the specific application. For instance, the use of nanoparticles as external contrast agents for MRI requires nanoparticles with high magnetization; but a small particle size is necessary if they must escape from the mononuclear phagocyte system (MPS). Hence, superparamagnetic nanoparticles may be the answer. For hyperthermia therapy, ferromagnetic nanoparticles with a large area enclosed by their hysteresis loop are the most effective. The potential for magnetic manipulation can be the most important property of nanoparticles used as translational vectors in biological applications, such as drug delivery [12] and mechanical cells [13]. The force of attraction that can be exerted on MNPs by an external field is given by the equation:

(2) $${\vec{F}}_{m}=V\left({\vec{M}}_{s}\cdot \nabla \right)\vec{B}$$

where *F*_m_ is the magnetic force experienced by the particle, *V* the particle volume and *B* the magnetic field intensity. Since, through the volume, the force depends on size, large particles would be necessary to ensure a strong attractive force. However, the MNPs must be small enough to remain within the superparamagnetism regime.

## 3. Iron Oxide Nanoparticles

Many applications of MNPs still rely on the use of iron oxide particles. Iron oxide can exist in different chemical compositions, such as magnetite (Fe_3_O_4_) or maghemite (γ-Fe_2_O_3_), or, most commonly, a non-stoichiometric combination of the two. Magnetite and maghemite are by far the materials most frequently used for biomedical applications, and therefore, this review focuses on them. The other iron oxides are the ferrites, whose general formula is MFe_2_O_4_ (M = Mn, Zn, Co, Ni).

Magnetite has a cubic inverse spinel structure with a face-centered cubic (fcc) unit cell (JCPDS 19-629) composed of 32 O anions, 16 Fe(III) cations and 8 Fe(II) cations. Half of the Fe(III) cations are tetrahedrally (tet) coordinated, while the other half and all of the Fe(II) ions are octahedrally (oct) coordinated, resulting in a unit cell of (Fe_8_^3+^)_tet_(Fe_8_^3+^ Fe_8_^2+^)_oct_O_32_ [14]. Magnetite can be subject to oxidation because of the reduced iron in the crystalline lattice. In the presence of oxygen, magnetite oxidizes to maghemite. In this process, Fe^2+^_oct_ is oxidized to Fe^3+^_oct_, resulting in vacancies confined to the octahedral sites. Accounting explicitly for these vacancies, the unit cell for maghemite (JCPDS 39-1346) can be written as (Fe_8_^3+^)_tet_(Fe_40/3_^3+^ □_8/3_)_oct_O_32_, where □ refers to a vacant site. It has been suggested that an outer coating of maghemite may develop at the particle-water interface during magnetite oxidation [15]. Synthetic maghemite often displays superstructure forms, which arise as a result of the cations and the vacancy ordering. In that case, the crystalline system changes from cubic to tetragonal (JCPDS 25-1402). The extent of vacancy ordering is related to both the crystallite size and the amount of Fe(II), or other impurities, in the structure [16]. These possible maghemite arrangements are partially responsible for the differences in magnetic behavior manifested by maghemite nanoparticles prepared via different routes [17].

Because the crystalline structures of magnetite and maghemite are nearly identical, the two oxides have very similar physical properties. For instance, the cell parameter, *a*, is 0.8394 nm for magnetite and 0.8346 nm for maghemite. The difference is due to the Fe(II) ions in the structure of magnetite having a higher ionic radius than the corresponding Fe(III) ions. Both oxides are considered to be ferromagnetic, although magnetite has a larger bulk *M*_s_ (92–100 emu/g) than maghemite (*M*_s_ = 60–80 emu/g) and a lower Curie temperature (*T*_C_,_magnetite_ = 850 K, *T*_C_,_maghemite_ = 948 K), due to antiparallel interactions between the electron spins of tetrahedrally-coordinated Fe^3+^ and octahedrally-coordinated Fe^3+^/Fe^2+^ in magnetite [18,19]. Values of *M*_s_ depend on the size of the particles: *M*_s_ decreases as the volume of the particles decreases, although the variation is higher when the particles are less than 9 nm in diameter, due to the magnetically-inactive first atomic layer at the surface of the particles. This layer, which also exists in bulk materials, has a non-negligible volume in small particles. When the size is greater than 9 nm, *M*_s_ is close to the value of the corresponding bulk material, especially at sizes greater than from 14 nm, when it reaches 90% of the corresponding value for bulk ferromagnetic material at room temperature [20].

Below certain sizes, both oxides exhibit superparamagnetic behavior, although their *D*_SPM_ and *D*_CR_values are slightly different: 25 nm (magnetite) and 30 nm (maghemite) for *D*_SPM_, and 128 nm (magnetite) and 166 nm (maghemite) for *D*_CR_ [21] (Figure 3).

These two compounds fulfill the prerequisites of: (1) chemical stability under physiological conditions; (2) low toxicity; and (3) sufficiently high magnetic moments [22].

IONs tend to be classed as superparamagnetic oxides (SPIO), if the individual particles (size of core/shell) are larger than 50 nm, or ultra-small superparamagnetic iron oxides (USPIO), for particles smaller than 50 nm.

## 4. Magnetically-Guided Drug Targeting

Since the pioneering idea proposed by Freeman *et al.* [23] that fine iron particles could be transported through the vascular system and be concentrated at a particular point in the body with the aid of a magnetic field, the use of magnetic particles for the delivery of drugs or antibodies to organs or tissues altered by disease has become an active and attractive field of research [24]. The method of magnetically-guided drug targeting (MGDT) involves: first, the immobilization of a drug in MNPs; then, the injection of the drug/carrier complex into the subject, either via intravenous (i.v.) or intra-arterial (i.a.) injection; and, finally, the use of high-gradient external magnetic fields generated by rare-earth permanent magnets (generally NdFeB magnets with a maximum surface flux density of a little over one T) to guide the complex and concentrate it at the desired locations. Once the complex is concentrated at the target *in vivo*, the therapeutic agent is then released from the magnetic carrier, either via enzyme activity or through changes in physiological conditions, such as pH, osmolality or temperature. This results in increased uptake of the drug by the tumor cells at the target sites [25] and a limited systemic drug concentration [26].

The development of magnetically-guided nanoparticles has allowed the usual accumulation of nanoparticles observed in particular pathologies, such as tumors, inflammatory and infectious sites, to be enhanced. Those pathologies are characterized by structural abnormalities in the vasculature. This phenomenon, known as the enhanced permeability and retention (EPR) effect [27], is a consequence of the affected tissues possessing “leaky” vasculature, which allows macromolecules and nanoparticles to extravasate and accumulate more readily.

The method of MGDT is not only dependent on physical properties, concentrations and the amount of particles applied, but also on the type of binding of the drugs. In addition, the geometry, strength and duration of the external magnetic field, as well as the route of ION injection and the vascular supply to the targeted tissues will all influence the effects. The physiological parameters of the patient, such as body weight, blood volume, cardiac output, peripheral resistance of the circulatory system and organ function, will also affect the efficiency of the external magnet; apart from the possibility of placing the magnet in close vicinity to the target location [28]. To sum up, the process of drug localization using magnetic delivery systems is based on the competing forces exerted on the particles by the blood compartment and by the magnetic force generated by the external magnetic field (Equation (2)). Moreover, the administration route of the MNPs seems to be crucial for the success of the therapy, since i.a. delivery avoids, or at least minimizes, the particle clearance by the mononuclear phagocyte system (MPS) in liver and spleen in comparison to intravenously-applied particles [29]. Preliminary theoretical studies of the hydrodynamic conditions of MNP targeting and estimations from experimental work indicate that for most magnetic carriers, the field strength (flux density) at the target site should be of the order of 200–700 mT with gradients along the *z*-axis of approximately 8–100 T/m, depending on the flow rate (higher blood flow rates require either stronger fields or higher gradients) [30,31]. As a general rule, the model indicates that when the magnetic forces exceed the linear blood flow rates in arteries (10 cm·s^−1^) or capillaries (0.05 cm·s^−1^) [17], the MNPs will be retained at the target site and may be internalized by the endothelial cells of the target tissue. Moreover, the results derived from the model indicate that magnetic targeting is likely to be more effective for targets that are near the surface and in regions of slower blood flow. However, the model is somewhat idealized and limited; a more recent model has been developed in which particle capture is modeled for a variety of field/particle configurations in a two-dimensional branching network [32].

The newer model also incorporates the effects of sheer-induced diffusion due to the presence of cells within the blood plasma. The results indicate that it will not be possible to target a specific site *in vivo* without some degree of distribution to the surrounding tissue. For this reason, the authors [32] also conclude that magnetic targeting will probably be most effective for targets that are close to the surface of the body.

Although the use of permanent neodymium iron boron magnets in combination with IONs with excellent magnetic properties allow the depth of the magnetic field achieved to be increased up to 10–15 cm [24], the existence of physical constraints on magnetic targeting, such as the rapid drop off of field strength with target depth within the body and the difficulties of bypassing intervening vasculature and tissue structures [25,33], have hampered the clinical realization of this technology. Much of the recent work in this area has focused on the development of high-moment MNP carriers with novel, multifunctional coatings and novel techniques for enhancing the body’s own targeting systems [34]. Furthermore, progress is being made in improving the delivery of magnetic forces via magnetic needles, meshes and bandages, as well as through new methods for creating “stealth” delivery vehicles that use magnetic particles incorporated in macrophages or stem cells. One of these improvements is the approach referred to as magnetic resonance navigation (MRN), which has been proposed to steer and track in real time endovascular magnetic carriers in deep tissues to target areas of interest [35,36,37,38] and to restrain the systemic carrier distribution. MRN is achieved with a clinical MRI scanner upgraded with an insert of steering coils [38,39]. The scanner allows tracking the carrier during MRN along a pre-planned trajectory. The magnetic field (1.5 T or higher) of the system enables *M*_s_ of ferromagnetic materials throughout the body [35,37]. Hence, the problem of a weaker magnetic field in deep tissues observed with an external magnet can be overcome.

A good model for simulating the magnetic properties of MNPs in blood vessels is the “magnetic field capture of IONs in flowing systems”. The system consists of a circuit of rubber tubes, pumps and magnets, together with a gaussmeter to measure the strength of the magnetic field. It makes it possible to simulate the distance necessary to hold and accumulate IONs in moving fluid systems with different viscosities (water, glycerol 30%) [28]. An important *in vitro* study with regard to the simulation of the effect of magnetic particles on blood vessels was that carried out by Seliger *et al.* [40]. They made a circulating system consisting of a circuit with an intact bovine femoral artery close to an external magnetic field. After applying the nanoparticle suspensions by a side inlet, they observed that the accumulation of nanoparticles and the drug in the target region was higher after the i.a. application compared to the i.v. application.

### 4.1. In Vitro Studies

Kellering *et al.* [41] used an array of permanent magnets consisting of cylindrical NdFeB magnets of 4.5 mm diameter arranged with alternating polarity. Flasks containing breast carcinoma cells were placed on the magnets, and starch-coated IONs were added. After incubation, the extent of the magnetically-induced cell labeling was determined by measuring the iron content of control and labeled cells. The results showed that there was selective, higher MNP accumulation within tumor cells, due to the effect of the magnets. Along similar lines, Hardiansyah *et al.* combined chemotherapy and thermotherapy using doxorubicin (DOX)-loaded magnetoliposomes (MLPs) and colorectal cancer cells (CT-26 cells) [42].

Antioxidant enzymes are a promising therapeutic option for pathological conditions involving increased production of reactive oxygen species (ROS). However, their efficiency in combating oxidative stress is dependent on the ability to achieve therapeutically adequate levels of active enzymes at the site of the ROS-mediated injury. Thus, the implementation of antioxidant enzyme therapy requires a strategy both for guided delivery to the target site and effective protection of the protein in its active form. The utility and therapeutic potential of MNPs for site-specific delivery of biologically-active enzymes has remained largely unexplored due to the not inconsiderable challenges involved in the design of such formulations. To address those requirements, Chorny *et al.* developed a magnetic force-triggered protein delivery system based on MNPs prepared via the precipitation of calcium oleate in the presence of magnetite-based ferrofluid [43]. This system displayed great efficiency in the encapsulation of antioxidant enzymes, such as catalase and superoxide dismutase. The authors hypothesized that catalase-loaded MNPs applied with a high-gradient magnetic field could rescue bovine aortic endothelial cells from hydrogen peroxide toxicity in culture. Their results showed that the approach was not only capable of achieving the loading of therapeutically adequate amounts of protein without compromising the biological activity, but it could also create a sub-micron-sized, biocompatible carrier structure that could provide protection from potential proteolytic deactivation, while allowing permeability for the enzyme substrate.

Cho *et al.* developed a magnetic switch for the control of cell death signaling [44]. In that work, death receptor 4 (DR4) monoclonal antibodies highly expressed on tumor cells were conjugated to MNPs (zinc-doped IONs) via a specific antigen-antibody interaction. When applying a magnetic field to the aggregate MNP-conjugated DR4s, the magnetic switch is “on”, thus promoting apoptosis signaling pathways. The efficacy of the magnetic switch in inducing apoptosis *in vivo* was evaluated using a zebrafish model. An approximately 3.5-fold morphological alteration in the tail region, which was a visible consequence of apoptosis signaling, confirmed the occurrence of apoptosis after applying a 0.50 T magnetic field for 24 h.

Chen *et al.* prepared a nanoplatform for guided drug delivery by conjugating quantum dots with carbon nanotubes filled with magnetite. The platform was capable of transporting DOX into HeLa cells by means of an external magnetic field [45].

Chiang *et al.* developed DOX-loaded hollow hybrid nanogels prepared by the coassembly of IONs with the graft copolymer comprising acrylic acid and 2-methacryloethyl acrylate units as the backbone and poly(ethylene) glycol and poly(*N*-isopropylacrylamide) as the grafts. With magnetic transport guidance toward the target and subsequent exposure to an alternating magnetic field (AMF), this nanogel system showed superior cytotoxicity against HeLa cells than free DOX did [46].

All of these studies were carried out in 2D cell cultures, and they demonstrate that an external magnetic field can assist in the cellular uptake of drug-loaded nanoparticles. Child *et al.* performed a study to establish whether the potential for the combined use of a magnetic field and penetration can be successfully applied to cells growing in a 3D environment (a fibroblast-seeded 3D collagen gel). Their results showed that magnetic field and cell-penetrating peptides form an excellent partnership to achieve therapeutic drug delivery *in vivo* [47].

Another application of the effect of MLPs on cells is a kind of tissue engineering methodology. For instance, it is possible to culture keratinocytes and to use them to reconstitute human tissue [48,49]. Ito *et al.* showed that keratinocytes labeled with cationic MLPs were accumulated through the use of a magnet, and stratification was promoted by a magnetic force, thus forming a sheet-like 3D construct. The addition of cationic MLPs to human keratinocytes resulted in the rapid uptake of magnetite nanoparticles, and the MLPs accumulated in the keratinocytes reached a maximum of 70% of all of the MLPs added [50]. Other studies confirmed that this technique is a promising approach for tissue engineering [51,52,53].

More recently, with the aim of overcoming the problems posed by the blood-brain barrier when it comes to the transport of drugs into the brain, Thomsen *et al.* [54] used IONs as drug carriers. The IONs were shown to have a significantly increased capacity to penetrate the barrier (human brain microvascular endothelial cells) in the presence of an external magnetic force.

### 4.2. In Vivo Studies: Animal Models

#### 4.2.1. Chemotherapy

**Figure 4:**
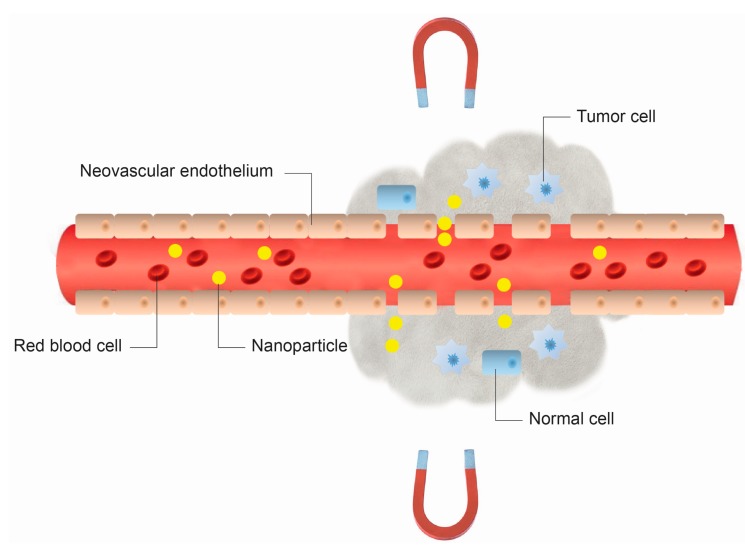
Schematic representation of the method of magnetically-guided drug targeting. Nanoparticles extravasate from pores in leaky tumor blood vessel walls (*i.e*., enhanced permeability and retention (EPR) effect).

Nowadays, one of the most active fields of biomedical research into cancer therapy is the design of new anti-tumor devices, and within this context, biocompatible MNPs have innumerous applications [55]. The major disadvantage of most chemotherapeutic approaches to cancer treatment is that they are non-specific. The i.v. administration of therapeutic (generally cytotoxic) drugs leads to general systemic distribution. The non-specific nature of this technique results in the well-known side effects of chemotherapy, as the cytotoxic drug attacks normal, healthy cells in addition to its primary target: tumor cells. Therefore, MGDT is seen as a potential means of increasing the efficacy and reducing the unpleasant side effects associated with chemotherapy, by reducing systemic distribution in combination with the possibility of administering lower, but more accurately targeted doses of the cytotoxic compounds used in these treatments (Figure 4).

One of the first studies of magnetic targeting of chemotherapy involved the use of a carrier formed of albumin microspheres (of several μm in diameter) with entrapped Fe_3_O_4_ and DOX that could be concentrated at a predetermined site *in vivo* by a magnetic field. This delivery system allowed for the local accumulation of DOX comparable to that achieved by administration of a 100-fold higher dose of the free drug [56]. A flood of studies concerning similar drugs using animal models (pigs, rabbits and rats) were then reported by several groups, following initial encouraging results [24,57,58,59,60,61,62,63,64,65,66,67]. In 1996, Lübbe *et al.* [68] used IONs coated with starch, to which 4'-epidoxorubicin was chemically bound. The drug/carrier was administered via i.v. injection to nude mice and rats and directed into the tumor using a magnetic field (permanent magnet; magnetic field strength, 0.5–0.8 T). The drug/carrier was well tolerated, and tumor remission was achieved.

Pulfer and Gallo [69] demonstrated that magnetic microspheres (1–2 μm in diameter) could be concentrated at the site of intracerebral rat glioma tumors. Although the concentration of the particles in the tumor was low, it was significantly higher than that of non-magnetic particles. In a later study, the group demonstrated that 10–20 nm IONs were more effectively targeted at these tumors in rats. Electron microscopy analysis of brain tissue samples revealed the presence of magnetic carriers in the interstitial space in the tumors, but in normal brain tissue, they were only found in the vasculature [70]. Mykhaylyk *et al.* [71] had less success using magnetite-dextran nanoparticles, but were able to target rat glial tumors by disrupting the blood-brain barrier immediately prior to particle injection.

Magnetoliposomes are a more suitable carrier to transport and deliver a drug, especially due to the smaller size of liposomes compared to albumin microspheres. Magnetoliposomes have a typical core/shell structure: a magnetic iron oxide core surrounded by an artificial liposome. Viroonchatapan *et al.* [72] designed a study to determine whether dextran-IONs incorporated into liposomes and containing calcein as a fluorescent marker could be targeted at mouse liver with the aid of an extracorporeal magnet. The study consisted of an on-line liver perfusion system with a fluorescence detector. The targeting efficiency was higher in the presence of a magnetic field.

As mentioned above, the capture of magnetic particles at a target as a consequence of a magnetic field is effective for targets close to the body’s surface; but the magnetic field strength falls off rapidly with distance, so sites deeper within the body become more difficult to target. Some groups proposed a way around this problem by implanting magnets near the target site, within the body [73,74]. In another study, an increase in anti-tumor activity and the elimination of weight-loss as a side effect were reported [75]. Similar results were obtained in yet other studies [76].

Alexiou *et al.* [77] treated squamous cell carcinoma in rabbits with mitoxantrone (MTX) bound to phosphate groups of MNPs coated with starch derivatives. When the implanted carcinoma had reached a volume of ≈3500 mm^3^, the MNP-MTX was administered via i.a. (femoral artery) or i.v. (ear vein) injection, while an external magnetic field was focused on the tumor. The i.a. administration of the complex resulted in a significant (*p* < 0.05), complete and permanent remission of the carcinoma in comparison with both the control group (no treatment) and the i.v. group. Typically, 35 days after the treatment, the tumor disappeared completely. In addition to this, the dose of the drug applied could be diminished to just 20% of the regular systemic dose. No metastases or negative side effects were observed. An important advantage of these carriers is the possibility of detecting the nanoparticles after treatment with common imaging techniques (e.g., X-ray tomography, magnetorelaxometry, MRI), which can be correlated to histology. Studies of the distribution of the particles showed a high accumulation of magnetic particles in the tumor region, in contrast to other body compartments (liver or spleen). In experiments using radioactive ^59^Fe-nanoparticles, 114-times more activity was detected in the tumor region after MGDT than in the control without a magnetic field [78]. Later, the same group observed that the magnetic field caused the drug not only to concentrate in the cancer tissue, but also to penetrate the tumor cells. The chemoadsorptive binding was strong enough to be stable during the transport process. Desorption occurred with a half-life of about 30 min and released the drug in its active form [79]. More recently, Tietze *et al.* performed a study comparing the biodistribution and therapeutic effects of nanoparticle-bound and unbound MTX after i.v. or i.a. administration in rabbits. The study showed the advantages of MGDT over the classic chemotherapy [80].

Paclitaxel has been one of the most important chemotherapeutic agents against cancer for several decades [81]. The drug has been formulated in a vehicle composed of a 50:50 (*v*/*v*) blend of Cremophor EL (polyethoxylated castor oil) and ethanol. However, the formulation induces histamine release and severe allergic reactions [82]. To avoid these undesired effects, paclitaxel has more recently been encapsulated in liposomes, with good results both *in vitro* and *in vivo* [83]. After i.v. or intraperitoneal administration, the paclitaxel-liposome formulations are much better tolerated than paclitaxel in the previous vehicle and showed antitumor activity similar to that of the drug in the Cremophor EL/ethanol modality. However, liposomes are subject to opsonic phagocytosis by circulating phagocytes and by macrophages in liver and spleen. Zhang *et al.* used magnetoliposomes as a strategy to overcome this problem [84]. The formulation was administered via i.v. injection to mice previously inoculated with breast tumor cells. Magnetoliposomes were guided and retained at the target site with the help of the magnetic field from a circular permanent magnet placed directly on the skin surrounding the tumor mass, via a sterilized rubberized fabric. The results showed that paclitaxel-magnetoliposomes could effectively be delivered to the tumor and exerted significant anticancer activity with fewer side effects.

Béalle *et al.* prepared non-drug-encapsulating magnetoliposomes. In this case, IONs were encapsulated in a volume fraction of up to 30%. The MNPs were rapidly and efficiently internalized by cultured tumor cells, and when they were administered to mice, they could be guided to tumors by an external magnet [85].

Chertok *et al.* used IONs coated with starch or gum arabic modified with polyethyleneimine (PEI) as a potential vascular drug/gene carrier for delivery to brain tumors [86]. The MNPs were injected into rats harboring orthotopic gliosarcoma. The intra-carotid administration route was shown to be optimal. Although no drug was administered, the study demonstrated that the surface modification with polycationic PEI resulted in 5.2-fold higher tumor accumulation of PEI-MNPs compared to uncoated MNPs.

**Figure 5:**
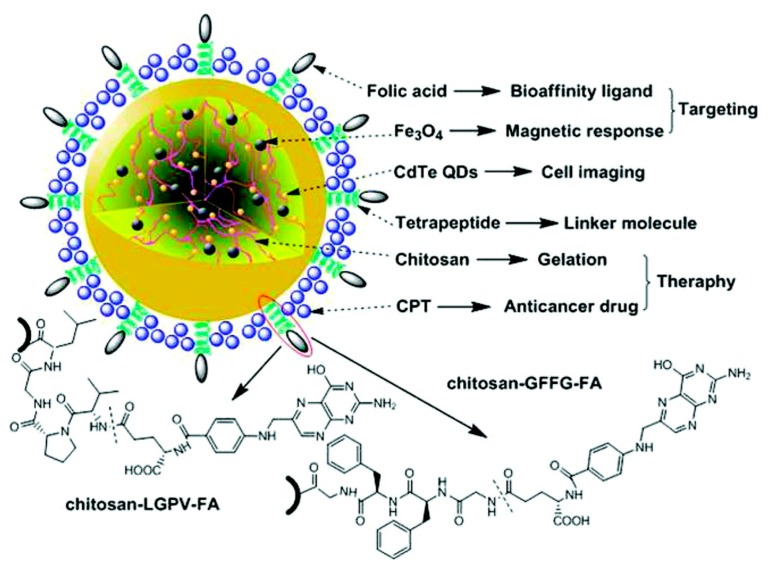
Schematic diagram of the design of multifunctional nanoparticles-tetrapeptide folic acid copolymers and their potential applications in biomedicine. GFFG and LGPV are the tetrapeptides used as linker molecules (in the single-letter amino acid code: GFFG = Gly-Phe-Phe-Gly; LGPV = Leu-Gly-Pro-Val) (reproduced with permission from [87], Copyright 2012, American Chemical Society). QD, quantum dot.

Another kind of MNPs is based on gel combinations. Shen *et al.* synthesized luminescent MNPs with folate-conjugated tetrapeptide composites by *in situ* assembly for targeted drug delivery to tumors (Figure 5). The particles contained: camptothecin (CPT), a broad-spectrum anticancer agent; CdTe quantum dots (QDs), a fluorescent dye; magnetite; folic acid, a target ligand with a high binding affinity for folate receptors, which overexpresses on the surface of many human malignant cell membranes; and finally, chitosan as the gelation material to coat the magnetite and QDs. First, the chitosan, QDs and magnetite were directly gelled into ternary hybrid nanogels. Subsequently, the tetrapeptides and folate were conjugated in an orderly fashion with the hybrid nanoparticles [87].

The resulting MNPs were injected into sarcoma-bearing mice. It was demonstrated that the MNPs were trapped in the tumor tissue under external magnetic guidance. Additionally, the study confirmed that these MNPs inhibit the growth of cancer cells more than that of normal cells.

Pouponneau *et al.* have used the MRN approach to improve drug targeting in deep tissues [88]. They used microparticles loaded with iron-cobalt nanoparticles and DOX in rabbits. Rabbits were placed in the tunnel of a clinical MRI scanner, and the imaging gradient coils were used to move the MNPs-based carriers along a pre-programmed path in the vasculature towards the target site. By performing such targeting in the tunnel of a scanner, the MNPs were at saturation magnetization at any depth and, hence, did not suffer from the rapid decay that occurs with a permanent magnet placed at the surface.

#### 4.2.2. Other Biomedical Applications

Magnetic targeting was also used to break up thrombi. Thrombosis was induced in dog and rabbit arteries by surgically inverting a vascular wall flap into the lumen. Once the thrombus had been formed, a SmCo magnet was externally secured to one of the arteries. The magnetic field produced by the magnet had no effect on the clot formation. Autologous red blood cells loaded with a ferromagnetic colloid compound and aspirin were administered intravenously and completely reversed the arteriothrombosis in the artery under the effects of the magnet; whereas there was no alteration on clot formation in a control artery [89]. Torchilin *et al.* [90] obtained streptokinase immobilized on dextran-coated microparticles of iron oxide, and used this drug/carrier for targeted thrombus lysis in dog carotid arteries. After inducing the formation of a thrombus in matching arteries on each side of a dog, a small permanent SmCo magnet was implanted into the tissue next to one of the vessels, in the region of the vascular surgery and potential thrombus formation. After administration of the thrombolytic preparation, blood flow almost completely recovered in the artery with the magnet.

Integrins are transmembrane receptors that act as cell adhesion molecules. One integrin, α_V_β_3_-integrin, is over-expressed in tumor vasculature and invasive tumor cells. A ligand for this integrin is the peptide arginine-glycine-aspartic acid (RGD, in the single-letter amino acid code) [91]. Coupling RGD onto the surface of any particle facilitates the targeting of tumors and sites of inflammation (rich in integrin molecules). Jain *et al.* [92] used negatively-charged magnetoliposomes, some of them coated with RGD peptide and others encapsulating the anti-inflammatory drug diclofenac. They conducted an *ex vivo* study (uptake of neutrophils and monocytes; cells that also express integrin receptors) and an *in vivo* study in rats. It was observed that magnetoliposomes were selectively taken up by the circulating blood monocytes/neutrophils, thereby averting uptake by fixed macrophages in the liver. Coating the liposomes with RGD peptide further increased their uptake via receptor-mediated endocytosis. This uptake, in turn, imparts magnetic properties on these cells, and because of their tendency to migrate towards inflammatory sites and under the guidance of an external magnetic field, the drug can actively be targeted at any poorly accessible site of inflammation, e.g., brain. Such systems could be useful in the treatment of neurological diseases, such as Alzheimer’s disease, Parkinson’s disease, prion disease, meningitis, encephalitis, brain tumors, *etc.*, with an inflammatory component.

**Figure 6:**
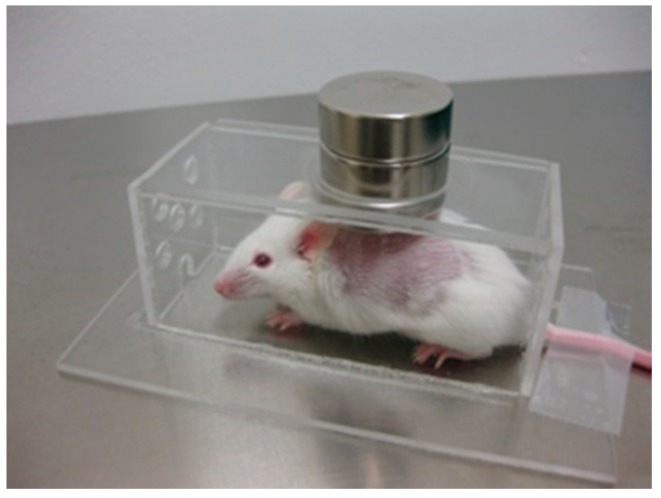
Experimental device to determine the effect of an external magnetic field on the biodistribution of MNPs after i.v. injection into mice in which an inflammatory focus had been induced on their backs. Two Nd_2_Fe_12_B disk magnets, 25 × 10 mm, of 600 mT per side were placed over the hole in the Plexiglas cages.

Magnetic targeting can also be used to treat inflammatory processes. In an *in vivo* study, magnetoliposomes were administrated via i.v. injection to mice in which an inflammatory focus had been induced on their backs. As in cancer pathologies, nanoparticles passively concentrated at the inflammation zone by means of the EPR effect. However, when an external magnetic field was applied near the inflammation (Figure 6), that higher amounts of iron were observed in the exudates, while the liver, spleen and plasma showed a lower iron concentration than in the absence of the magnet [93]. This suggested that the magnet efficiently retained the magnetoliposomes in the inflammation zone.

### 4.3. In Vivo Studies in Humans

As indicated above, numerous small animal studies have been reported. However, due to the technical difficulties mentioned (rapidly diminishing field strength with target depth in the body and the difficulties of bypassing intervening vasculature and tissue structures), clinical applications largely remain a future goal [34]. To date, no application of magnetic targeting in humans has reached the marketplace; although some phase I/II clinical trials have been conducted. Lübbe *et al.* reported the first worldwide clinical trials using MGDT [94]. IONs were coated with starch and the resultant complex bound to epirubicin through binding the positively-charged amino sugars of epirubicin to the anionic phosphate groups of the starch. The trial consisted of i.v. infusion of a chemically-bound drug and one course of conventional chemotherapy. During infusion, and for 45 min afterwards, a magnetic field was built up as close to the advanced (and unsuccessfully pre-treated) tumor as possible. Though the particles accumulated in the liver, the study demonstrated that magnetic carriers were generally well tolerated. However, despite the promise of MGDT, there are still several hurdles to overcome prior to achieving success in humans, including poor targeting in deep tissue (*i.e*., >2 cm depth) and poor retention of the particles upon removal of the external field [95].

At the beginning of 2002, a pharmaceutical company designed magnetic targeted carriers (MTCs) composed of elemental iron particles and activated carbon, to deliver drugs to liver cancer patients. The MTCs (1–2 µm in size) could adsorb and desorb drugs, such as DOX. A mix of the MTCs and DOX was injected into an artery near the tumor, followed by the application of a powerful magnetic field to aid the localization and retention of the particles at the targeted site by extravasation of the particles into the surrounding tissue. The magnetic field caused the drug to be released into the tumor. When the magnetic field was turned off, a high percentage of the particles remained trapped in the tumor, and only a small percentage of the drug circulated throughout the body. Since the total amount was very small, there were practically no side effects [26], and hence, the amount of drug necessary was much less than had it been dispersed throughout the whole body. However, after a phase III clinical trial, the company decided to stop enrolling patients when interim analysis revealed that the goal of the study, to increase survival, would not achieve statistical significance. MTCs labeled with β-emitters, such as rhenium-188 and yttrium-90, were also used in the treatment of liver and brain tumors [96].

In 2004, Wilson *et al.* published encouraging results of a clinical study combining magnetic targeting and MRI, in which they were able to monitor the trans-catheter delivery of magnetically-targeted DOX to the hepatic artery using intra-procedural MRI [97]. The study demonstrated that there was selective targeting of the tumor, with a final fraction of treated tumor volume of 0.64 to 0.91 compared with only 0.07 to 0.30 for normal liver tissue.

## 5. Magnetofection

The introduction of foreign nucleic acids (plasmid DNA or small interfering RNA (siRNA) constructs) into a eukaryotic cell is called transfection. There are two approaches to transfection: viral and non-viral. Although viral delivery is the more conventional approach, because viruses have evolved to infect cells with a high efficacy, it presents important safety risks. For this reason, for gene therapy, attention has focused on non-viral approaches, as these have the potential to overcome many of the inherent challenges of viral vectors. Originally, non-viral gene delivery was simply the delivery of naked plasmid DNA. To improve the stability and uptake of the DNA, physical strategies (e.g., hydrodynamic delivery or electroporation) and specific particles (*i.e*., cationic polymers, peptides, liposomes and solid-core nanoparticles) were developed. One method for attaching DNA to the surface of particles is to employ the electrostatic interactions between the negatively-charged phosphate backbone of DNA and positively-charged molecules linked to the particle surface. A popular choice for this method is the cationic polymer PEI, which facilitates lysosomal release of the complex following internalization by buffering the intralysosomal pH and, thereby, causing the lysosome to rupture and release its contents [98]. Since it is understood that particle DNA complexes typically enter a cell by endocytosis through clathrin-dependent pits [99], it is possible that this feature of PEI will benefit PEI-coated particles.

A special method of gene delivery is magnetic transfection, or “magnetofection”. Magnetofection is the delivery of nucleic acid under the influence of a magnetic field that acts on nucleic acid vectors that are associated with MNPs. Magnetofection works along similar physical principles to those of magnetic targeting. A high-field, high-gradient magnet is generally placed underneath a cell culture dish or multi-well plate. The particle-gene complex is introduced into the cell growth medium, and the magnetic field rapidly pulls the particles into contact with the cells growing on the bottom of the dish. The efficiency of magnetofection has been demonstrated, since it facilitates the introduction of nucleic acids into the nucleus multifold, compared to routinely available standard technologies [100].

The first accounts of magnetofection in the literature are conference abstracts by Mah *et al.* [101] and Plank *et al.* [102] in the year 2000. The first full paper in a scientific journal was by Hughes *et al.*, on magnetically-enhanced retroviral nucleic acid delivery [103], followed by the work by Scherer *et al.* [104] on non-viral and viral magnetofection and Mah *et al.* [105] on recombinant adeno-associated virus 2 delivery. Since this pioneering work, there has been a dramatic expansion in work aimed at adapting this technique for non-viral transfection of DNA or siRNA [106,107].

PEI-coated magnetic particles were first reported by Scherer *et al.* [104] and provided the first example of *in vitro* MNP-mediated non-viral gene delivery. Since that study, magnetofection has been used to transfect a number of cell types, including primary lung epithelial cells [108] and blood vessel endothelial cells [109]. Such particles have successfully been used to deliver antisense oligonucleotides [110] and siRNA to down-regulate gene expression. Schilinger *et al.* [99] reported that siRNA associated with magnetic particles significantly reduced retrovirally-mediated expression of luciferase in HeLa cells. McBain *et al.* [111] reported an alternative method for synthesizing PEI-coated magnetic particles based on covalently coupling PEI to the surface of composite iron oxide-dextran silica particles using glutaraldehyde linkers. As mentioned above, PEI has been shown to promote the endocytosis of particles, resulting in rapid and efficient transfection [112]. Several groups have also successfully employed non-viral nanomagnetic transfection to introduce siRNA into cells for gene knockout studies [113]. This involves attaching strands of siRNA to the particles. As the particles are taken into the cells, the siRNA blocks the activity of the target gene, knocking out its function. Such studies are particularly important for examining the specific genes involved in disease pathways.

Recently, further advances have been made by using oscillating magnet arrays placed beneath the culture dish, as well as pulsed electromagnets oriented perpendicular to the magnetization vector of the magnet below the culture dish [114,115]. The oscillations introduce a lateral component of motion into the particle-gene complex, which is superimposed on the *z*-axis motion due to the permanent magnet beneath the culture plate. This mechanical stimulation promotes more efficient endocytosis of the particle-gene complex and significantly increases transfection efficiency over that of other non-viral methods.

A novel approach examined by Stride *et al.* combines two physical transfection techniques: magnetofection and ultrasound [116]. The transfection efficiency of magnetic microbubble/nanoparticle complexes was found to be greater in Chinese hamster ovary cells when both the magnetic field and ultrasound were applied simultaneously. This interesting combination of methods may indicate future approaches to enhancing non-viral gene transfection, both *in vitro* and *in vivo*. As with MGDT, the development of magnetic targeting for *in vivo* gene delivery remains elusive. In a 2006 study, Xenariou *et al.* were not able to demonstrate gene transfection in a mouse model of cystic fibrosis [117]. However, in 2008, Hüttinger *et al.* published the results of a phase I trial of a veterinary application. The group showed that magnetofection was well tolerated as a potential gene therapy for feline fibrosarcomas [118]. Although the study aimed to evaluate toxicity, 10 of the 20 cats treated were recurrence-free after one year; which indicates a potential novel *in vivo* application of this technology. That study may point to a way forward in this area: refining approaches to drug delivery and gene therapy in the veterinary field first, as a stepping-stone towards human treatments.

Magnetofection has significant advantages over traditional transfection methods, including reduced process time (of the order of 10 min as opposed to 2–4 h), high transfection rates with lower vector doses and an increase in efficacy [119]. Moreover, magnetofection has the major safety advantage that it exploits natural uptake pathways (the endocytic mechanisms of cells during the transfection process, without disrupting the cell membrane), resulting in high cell viability post-transfection [120,121].

Magnetofection, as a strategy to improve the accumulation of gene delivery vehicles at the tumor site, works well for solid tumor masses, but it provides little advantage for highly invasive and infiltrative cancers, such as glioma, the most common and lethal type of brain cancers, since the cells of such cancers would not be accessible to a magnet. In contrast, chlorotoxin (CTX) is a promising targeting agent due to its capacity to specifically recognize a broad spectrum of cancers, including the vast majority of brain tumors. Kievit *et al.* developed a CTX-labeled magnetic nanovector by incorporating the targeting ligand CTX onto the surface of a nanovector comprised of an ION core coated with a copolymer of chitosan, PEG and PEI. The nanovector effectively bound to DNA. The targeted nanovector was injected into mice bearing C6 xenograft tumors. The uptake of the CTX-nanovectors in cancer cells was higher than that of the non-targeted vector [122].

The major benefits of magnetofection are an improvement in the dose-response relationship in nucleic acid delivery, a strong improvement of the kinetics of the delivery process and the possibility of localizing nucleic acid delivery in an area that is under the influence of a magnetic field.

## 6. Magnetically-Induced Drug Release

The first use of external magnetic fields to achieve pulsatile release from polymer composites was by Kost *et al.* [123], who observed externally-controlled insulin release from a magnetic composite of an ethylene-vinyl acetate copolymer by application of a low-frequency AMF. Even more interestingly, however, the inherent thermal energy from MNPs can be used as an external and remote-controlled trigger to control drug release. The energy can be used to open the gates for any kind of organic or inorganic carriers that contain drugs for therapy.

Although the first studies conducted to stimulate drug release applied microwave radiation (2.45 GHz) to liposomes with enwrapped ferromagnetic microparticles [124], the existence of some drawbacks (e.g., the particles presented limited biocompatibility due to their μm range and the surrounding tissue became substantially heated) meant that microwaves were substituted by a high-frequency (10–500 kHz) or low-frequency (<10 kHz) AMF and ferromagnetic materials by IONs. The AMF can suppress drug-drug carrier interactions and accelerate diffusion [125]. The drug release rate is significantly enhanced in the presence of a magnetic field, because the pulsatile mechanical deformation generates compressive and tensile stresses. Moreover, AMF-triggered drug delivery systems utilize the collapse or volume transition of the drug carriers to induce drug release [126].

Liposomes are the type of nanoparticles most commonly used for this purpose. Liposomes provide a way of dispersing and concentrating nanoparticles and/or drugs via encapsulation or binding, while shielding them from biomolecular adsorption. Therefore, nanoparticles or drugs can be delivered via established liposome delivery strategies. The simplest way for a drug to be released is by diffusion through the lipid bilayer. Diffusion is clearly greatest when the bilayer is disrupted or phase separated. Thus, the permeability of the bilayer influences drug diffusion, and the permeability of liposomes is greatly enhanced around the membrane melting temperature (*T*_m_) [127], which depends on the lipid composition. The cargo can thus be released if the liposome membrane is heated to above *T*_m_. For the use of liposomes as thermoresponsive drug delivery vehicles, *T*_m_ is typically designed to be close to body temperature, to release the cargo at the few degrees higher than the temperature of pathological tissue, such as cancer. However, under such circumstances, liposome leakage is produced during circulation. To circumvent the incompatible requirements of simultaneous release efficiency and low passive leakage, liposomes have been loaded and decorated with MNPs to trigger cargo release under the effect of a high-frequency AMF. Magnetoliposomes were the first multifunctional hybrid liposome/nanoparticle assembly and have received considerable attention since being introduced in 1988 [128].

One of first studies on controlled release from magnetoliposomes was performed by Viroonchatapan *et al.* [129]. Dextran-IONs incorporated in liposomes and containing calcein were prepared to study the temperature-dependent release of the fluorescent marker. The thermosensitivity and lipid integrity of the magnetoliposomes were not influenced by the inclusion of dextran-IONs. Later, Babincová *et al.* used magnetoliposomes encapsulating DOX and subjected them to the action of an AMF (≈1 MHz). The heat generated resulted in the release of the drug from the magnetoliposomes [130]. De Paoli *et al.* demonstrated enhanced dextran release by application of a low-frequency AMF to magnetic nanocomposites of collagen [131].

Tai *et al.* [132] entrapped dextran-coated IONs inside thermosensitive liposomes (the lipid composition was dipalmitoyl0phosphatidylcholine (DPPC), whose *T*_m_ is 41.2 °C). Carboxyfluorescein was used as a fluorescent probe to monitor the drug release induced by an AMF. The study was performed in solution, in phantom and in rat forearm skeletal muscle and demonstrated that the AMF triggered drug release.

To improve the sensitivity and responsiveness of MLPs, Chen *et al.* formed bilayer-decorated MLPs containing small hydrophobic SPIO nanoparticles: 5 nm maghemite capped with oleic acid (OA), embedded within lipid bilayers [133]. The heating of nanoparticles using alternating current electromagnetic fields (AC EMFs) operating at radio frequencies provided selective release of the encapsulated molecule at low nanoparticle concentrations and under physiologically-acceptable EMF conditions. Without radio-frequency heating, spontaneous leakage from the decorated MLPs decreased with increasing nanoparticle loading, consistent with greater bilayer stability and a decrease in the effective decorated MLP surface area due to aggregation. With radio-frequency heating, the initial rate and extent of leakage increased significantly as a function of nanoparticle loading and EMF strength. The mechanism of release is attributed to a combination of bilayer permeabilization and partial decorated MLPs rupture. The authors hypothesized that focused heating of embedded nanoparticles could be used to control and maximize leakage with minimal nanoparticle loading and EMF strengths. Moreover, nanoparticles can be embedded within lipid bilayers without significantly compromising the liposome structure.

**Figure 7:**
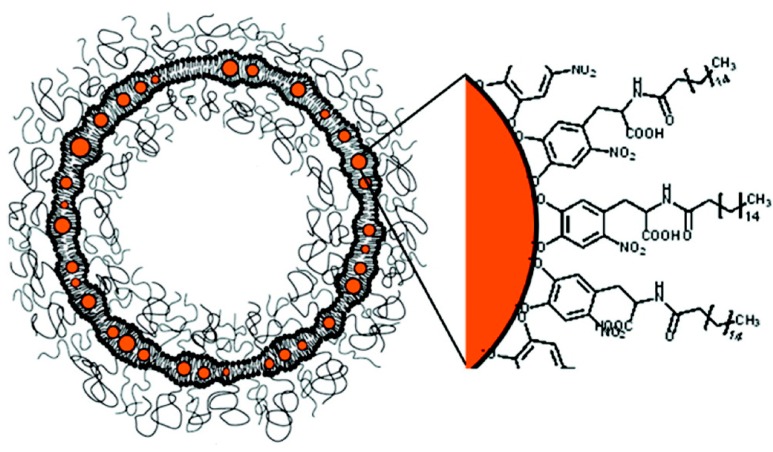
Schematic of liposomes containing iron oxide nanoparticles (IONs) in their bilayer. Palmityl-nitro-dihydroxyphenylalanine (DOPA)-stabilized IONs are embedded in liposome membranes consisting of PEGylated and unmodified lipids (reproduced with permission from [134], Copyright 2011, American Chemical Society).

Amstad *et al.* prepared [134] PEGylated liposomes with a *T*_m_ far higher than body temperature that hosted individually-stabilized IONs in their membranes in a well-defined structure (Figure 7). IONs with an average core diameter of 5 nm were surrounded by a palmityl-nitro dihydroxyphenylalanine (DOPA) shell with a dispersant packing density of ~1.5 palmityl-nitro-DOPA/nm^2^ and mixed with 2-distearoyl-sn-glycero-3-phosphocholine (DSPC) lipids containing 5 mol % 1,2-dioleoyl-sn-glycero-3-phosphoethanolamine-*N*-[methoxy(polyethylene glycol)-2000] (PEG (2)-PE). As a result, the liposomes were colloidally stable and impermeable at body temperature. Stable incorporation of IONs into liposome membranes allowed repeated, controlled release of the cargo, triggered by an AMF. The release efficiency was so high, that it was possible to choose AMF settings to release the cargo without increasing the bulk temperature to *T*_m_, even without external cooling.

To probe the permeation and actuation of liposomes containing IONs in their membranes, liposomes were loaded with the fluorescent probe calcein and then subjected to AMF pulses. After five subsequent 230-kHz AMF pulses, the fluorescence rapidly increased. Because the liposome structure was retained during the AMF treatment, the content could be released repeatedly and non-destructively from liposomes at bulk temperatures significantly below *T*_m_ of the liposomes. Consequently, the cargo could be released over prolonged periods, preventing bursts that can lead to temporary local overdoses.

More recently, Hardiansyah *et al.* have combined chemotherapy and thermotherapy using DOX-loaded MLPs and colorectal cancer cells (CT-26 cells) [42].

Gels are another system used as a stimulus-responsive platform. To enhance the influence of the external magnetic or electric fields on the gel properties, it is necessary to combine solid-like and fluid-like behavior. Therefore, new colloidal solutions termed “complex fluids” based on ferrofluids and polymer gels have been studied. Since polymer gels contain a substantial amount of liquid as a swelling agent, it is possible to design field-sensitive gels by using a polymer network swollen in a complex fluid. The colloidal particles incorporated within the gel, which are characterized by strong adsorptive interactions between solid particles and polymer chains, allow a fast response to an external field. These field-sensitive gels can be used to construct new types of controlled delivery systems. In this way, IONs have been embedded within hydrogels, and they can carry a therapeutic agent that is released upon heating [135,136]. It was observed that pulsatile release from magnetic nanocomposites of gelatin hydrogels was obtained by application of a high-frequency AMF [137].

Baeza *et al.* designed mesoporous silica nanoparticles with ION crystals encapsulated inside the silica matrix and decorated on the surface with a thermoresponsive copolymer of PEI-*b*-poly(*N*-isopropylacrylamide) (NIPAM) [138]. The nanocarrier traps the different cargos at low temperatures (20 °C) and releases the retained molecules when the temperature exceeds 35–40 °C. This increase in temperature can be achieved under the action of an AMF.

Devices based on nanocomposite membranes containing thermoresponsive nanogels (usually formed of NIPAM) and IONs can provide reversible, on/off drug release upon application (and removal) of an AMF. Hoare *et al.* demonstrated that the dose of drug delivered across the membrane can be tuned by engineering the phase transition temperature of the nanogel [139,140].

**Figure 8:**
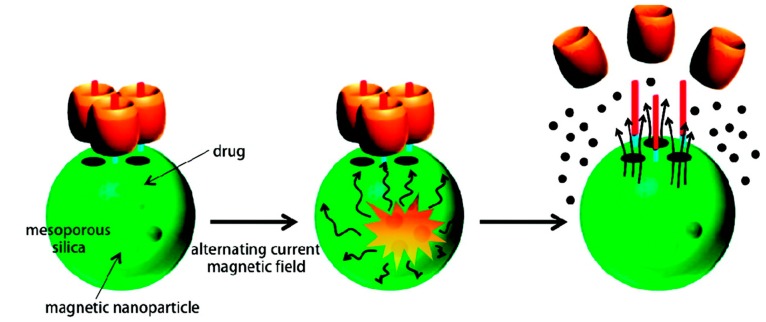
Magnetically-triggered drug release system. Schematics of nanoparticles, molecular machines, assembly and remote-controlled drug release. The particles and machines are not drawn to scale (reproduced with permission from [142], Copyright 2010, American Chemical Society).

Another strategy for the synthesis of magnetic/polymer nanoparticles involves the precipitation of magnetic IONs within a porous polymer microparticle or nanoparticle scaffold [141]. One advantage of this technique is that it is often possible to produce particles with a relatively narrow size distribution, as well as a well-defined spherical morphology. Specifically, 15 nm (Zn_0.4_Fe_0.6_)Fe_2_O_4_ nanoparticles have been incorporated into porous drug carrier nanoparticles with molecular valves [142]. A molecular valve, which consists of a thread and capping molecule (cucurbit[6]uril), closes the silica pores to keep the drug inside (Figure 8).

When an external AMF is applied, the generation of heat and subsequent buildup of pressure (~90 bar) inside the porous nanoparticles cause the rapid removal of the molecular valves and the release of the cargo. These materials are useful for *in vivo* drug delivery, as demonstrated by the release of DOX in breast cancer cells. MNPs were taken up by the cells, and minimal drug release was observed, because the valves attached to the surface were closed. In the presence of an AMF, the local heat caused by the MNPs facilitated the release of DOX from the silica pores, inducing apoptosis in the cells [142]. This result indicates that MNPs are effective as an actuator for controlled drug release from a carrier in a non-invasive and remote way.

A system that combines these two materials and a thermally-sensitive gatekeeper could constitute a unique drug delivery system. A novel material that incorporates zinc-doped IONs nanocrystals within a mesoporous silica framework that has been surface modified with pseudorotaxanes has been reported. Upon application of an AMF, the nanocrystals generate local internal heat, causing the molecular machines to disassemble and allowing the cargo (drugs) to be released. When breast cancer cells (MDA-MB-231) were treated with DOX-loaded particles and exposed to an AMF, cell death occurred. This material promises to be a non-invasive, externally-controlled drug delivery system with cancer-killing properties.

## 7. Conclusions

The development of MNPs has accelerated greatly in recent years due to advances in nanotechnology and molecular cell biology. Consequently, various formulations of MNP have been developed for theranostic applications. In particular, the use of MNPs as drug carriers has attracted enormous attention. However, in relation to magnetically-guided MNPs, efficient *in vivo* drug delivery is still elusive, with fundamental problems being the drop off in magnetic field strength with distance inside the body and ever smaller targets, such as individual nanoparticles, as well as the body’s physiological defense mechanisms against foreign agents. In contrast, the magnetically-guided approach has yielded clear benefits in genic transfection. Compared to all other physical methods, the major advantage of magnetofection is that it is capable of combining simplicity, a modest cost, enhanced localization and efficiency of delivery and reductions in both incubation time and vector doses. Promising *in vivo* results have also been reported for a pre-clinical trial of gene transfection in cats for the treatment of feline fibrosarcomas. Meanwhile, magnetically-sensitive MNPs constitute a platform that shows the highest diversity of innovations in the drug delivery field. Apart from the use of thermosensitive liposomes and gels that are sensitive to the heat generated by the action of alternating radiation on MNPs, other systems have yielded encouraging results.
